# Multidrug-Resistant *Campylobacer jejuni* on Swine Processing at a Slaughterhouse in Eastern Spain

**DOI:** 10.3390/ani11051339

**Published:** 2021-05-08

**Authors:** Clara Marin, Laura Lorenzo-Rebenaque, Judith Moreno-Moliner, Sandra Sevilla-Navarro, Estefania Montero, Mᵃ Carmen Chinillac, Jaume Jordá, Santiago Vega

**Affiliations:** 1 Departamento de Producción y Sanidad Animal, Salud Pública Veterinaria y Ciencia y Tecnología de los Alimentos, Instituto de Ciencias Biomédicas, Facultad de Veterinaria, Universidad Cardenal Herrera-CEU, CEU Universities, Calle Tirant lo Blanc, 7, 46115 Alfara del Patriarca, Spain; laura.lorenzorebenaque@uchceu.es (L.L.-R.); Judith.moreno@alumnos.uchceu.es (J.M.-M.); s.sevilla@cecav.org (S.S.-N.); estefania.montero@uchceu.es (E.M.); chinillachandreu@gmail.com (M.C.C.); jaume.jorda@uchceu.es (J.J.); svega@uchceu.es (S.V.); 2 Centro de Calidad Avícola y Alimentación Animal de la Comunidad Valenciana (CECAV), 12539 Castellón, Spain

**Keywords:** multidrug resistance, *Campylobacter*, swine, antimicrobial resistance, slaughterhouse

## Abstract

**Simple Summary:**

This study was designed to assess the epidemiology and antimicrobial resistance of *Campylobacter jejuni* in swine processing at a slaughterhouse in the Valencia Region (Eastern Spain). The results showed that all batches arrived at the slaughterhouse shedding *Campylobacter* in faeces, and remained positive during processing, even just before delivery to the consumer. In addition, 96.3% of *C. jejuni* isolates, the main species involved in human infection from food origin, were multidrug-resistant strains.

**Abstract:**

Campylobacteriosis is the most commonly reported gastrointestinal disease in humans in the EU, mainly from poultry meat consumption. *C. jejuni* is the main species involved in the human disease. However, little is known about the role of swine meat in its epidemiology. Thus, the aim of this study was to assess the epidemiology and antimicrobial resistance of *C. jejuni* on swine processing at the slaughterhouse. To this end, a total of 21 pig herds were intensively sampled at the slaughterhouse. *Campylobacter* isolation was based on official method ISO 10272-1:2018, speciation was determined by the hippurate hydrolysis test, and antibiotic susceptibility was performed according to standard disc diffusion assay. The results showed that all batches shed *Campylobacter* in faeces upon arrival at the slaughterhouse and remained positive at the end of the slaughtering process (42.8%). Moreover, 41.5% of *Campylobacter* strains isolated were *C. jejuni* and all of them were resistant to at least one antibiotic, and 96.3% were multidrug-resistant strains. In conclusion, the high level of multidrug-resistant *C. jejuni* swine batch contamination at the slaughterhouse makes it necessary to include the swine sector in national control programmes to reduce the bacterium and its resistance.

## 1. Introduction

Antimicrobial resistance (AMR) is one of the most important threats to public health worldwide [1]. While antimicrobial agents (AMAs) have been enormously beneficial since their peak in the mid-1950s, their inappropriate use led to AMR, one of the biggest global issues [2]. The World Health Organisation (WHO) published that by 2050, AMR will cause 10 million deaths and economic losses of $100 trillion annually [3]. In Europe, every year, 33,000 people die as a result of AMR [4]. Under the One Health concept, whereby livestock and agri-food systems are at the crossroads of human, animal, and environmental health, it is widely acknowledged that the use of antimicrobials in veterinary medicine has an impact on AMR transmission between farms, animals, and ultimately humans [5,6]. In this sense, the WHO published the priority list of 12 antibiotic-resistant bacteria, which includes *Campylobacter* [7].

Thermophilic *Campylobacter* is widely recognised as one of the major causes of foodborne illness worldwide, and the most commonly reported zoonotic pathogen in the European Union (EU), with approximately 220,000 confirmed cases occurring each year [8,9,10]. This situation is compounded by the fact that AMR *Campylobacter* strains emerge as a potential concern for public health safety, with implications of increased disease severity, longer hospitalisations, and higher cost rates [11,12]. In human outbreaks, *Campylobacter jejuni* (*C. jejuni*) is the main species involved, and pork is considered the major source of infection, after poultry meat [10,13]. 

Spain is the second largest swine producer in the EU and the fourth worldwide [14,15]. However, in this country, the swine industry administers approximately 75% of the AMAs used in veterinary medicine [16]. In the near future, an increase in the consumption of pork meat is expected; and a proper knowledge of the epidemiology of AMR *Campylobacter* in last steps of swine production chain is needed to control the bacteria [17,18]. Added to this, there is no legislation implemented in Europe for *Campylobacter* control in pork meat, in contrast to poultry production where, since 2018, the European Regulation (EC) No 2017/1495 has been implemented at poultry slaughterhouses to control the bacteria [19].

Studies have demonstrated that swine *Campylobacter* prevalence is higher at their arrival to the slaughterhouse than at farm level [17]. Thus, processing could increase this prevalence, especially the stages of scalding and evisceration, with the production of contaminated airborne droplets and the spilling of intestinal content, respectively [20,21,22,23,24,25]. The implementation of efficient measures at critical points at the slaughterhouse level could therefore improve control of the pathogen and its AMR in the final product [22,26,27]. However, it is difficult to implement proper control measures at slaughterhouse level if the epidemiology of *Campylobacter* in swine is unclear [28,29]. Traditionally, *C. jejuni* has been linked to food infection from chicken meat consumption [30], while *Campylobacter coli (C. coli)* has been linked to food infection by swine [31]. In this context, the aim of this study is to assess the epidemiology and AMR of *C. jejuni* in swine processing at the slaughterhouse.

## 2. Materials and Methods

Since only non-experimental clinical veterinary practices were performed and no handling of animals related to research was carried out, a formal ethics approval from the Welfare Body of the University CEU Cardenal Herrera with regard to the EU Directive 2010/63/EU was not required.

### 2.1. Study Design

This study was carried out in swine slaughterhouses in the Valencia Region (*n* = 8), Eastern Spain. The processing plants involved in this study slaughter 90% of the swine production in the Valencia Region [28]. During one year, 21 visits to the slaughterhouse were done to intensively sampling 21 different batches of swine. The batch was a group of swine coming from a single farm on a specific day. All farms were finishing farms, with swine of minimum age of 9 months and with an average live weight of 160 kg.

### 2.2. Sample Collection

From each batch sampled, faecal samples were aseptically collected, pooling faecal material (500 g) from five different points over the lairage pens at the slaughterhouse. It is important to highlight that cleaning and disinfection was carried out between batches; thus, the samples collected in pens were linked to a specific batch. Five swine were randomly selected from each batch. At the evisceration stage, the caecum from each animal selected was aseptically collected and placed into a sterile bag. To collect the caecal content, they were incised with a sterile scalpel blade and the content was placed in a sterile jar. Then, carcass samples were collected at two moments during processing: before and after cooling. To this end, a 100 cm^2^ area of the ham, belly, rump, and jowl from each swine selected were swabbed—rubbing a sterile swab (bioMerieux, Madrid, Spain) 10 times vertically and horizontally [28]. Moreover, environmental samples were taken from two sites (knives, whips) and from the slaughtering staff (operators) by vigorous swabbing of the surfaces with swabs. Finally, scalding water was collected directly into a sterile jar (1 L).

### 2.3. Campylobacter Isolation

Samples collected were tested by direct culture and enrichment culture based on official method ISO 10272-1:2018 for the isolation of *Campylobacter* [32]. In addition, faeces and caecal samples were analysed in parallel by direct culture. From the homogenised samples, direct culture was performed onto mCCDA agar (Modified Charcoal Cefoperazone Deoxycholate agar, Biolife, Sarasota, FL, USA) and Preston agar (AES-Biomerieux, Marcy-l’Etoile, France). The plates were incubated at 41.5 °C for 44 ± 4 h in modified atmosphere (5% O_2_, 85% N_2_, 10% CO_2_, CampyGen, Oxoid).

First, pre-enrichment of all samples was performed in Bolton broth (OXOID, Dardilly, France) (dilution 1:10). These samples were incubated at 37 °C for 5 ± 1 h and subsequently at 41.5 °C for 44 ± 1 h in a microaerobic atmosphere (5% O_2_, 85% N_2_, 10% CO_2_, CampyGen, Oxoid). After incubation, 100 µL of the pre-enriched broth were transferred to mCCDA agar and Preston agar (AES laboratories^®^, BruzCedex, France). These plates were incubated at 41.5 °C for 44 ± 4 h in microaerobic atmosphere. The confirmation of *Campylobacter* was performed by a mobility test using a dark field microscope, biochemical tests of oxidase and catalase, and streaking at different temperatures and atmospheres on Columbia Blood Agar (AES laboratories^®^, BruzCedex, France). The isolates were stored in duplicate at −80 °C for future speciation.

### 2.4. Campylobacter Speciation

All strains isolated were unfrozen and revived (Columbia Blood Agar, Oxoid Ltd., England, UK). Plates were incubated at 41.5 °C for 44 ± 4 h in modified atmosphere (5% O_2_, 85% N_2_, 10% CO_2_, CampyGen, Oxoid, England, UK). Finally, the hippurate hydrolysis test (Oxoid, Madrid, Spain) was used to determine the species of the *Campylobacter* [33,34]. 

### 2.5. Antimicrobial Susceptibility

*C. jejuni* strains isolated were unfrozen and revived (Columbia Blood Agar (Oxoid Ltd., England, UK). Plates were incubated at 41.5 °C for 44 ± 4 h in modified atmosphere (5% O_2_, 85% N_2_, 10% CO_2_, CampyGen, Oxoid, England, UK). The antimicrobial resistance was evaluated by a standard disc diffusion assay in Müeller-Hinton Agar medium (Scharlau, Barcelona, Spain) enriched with 5% defibrinated horse blood (Oxoid Ltd., England, UK). The turbidity of the inoculums of each isolate was adjusted to a concentration of 2.0 on the McFarland scale, and they were incubated at 41.5 ± 1 °C for 44 ± 4 h, specific for Thermophilic *Campylobacter*, under microaerobic atmosphere (84% N_2_, 10% CO_2_ and 6% O_2_) (CampyGen, Oxoid Ltd., England, UK) [35]. *C. jejuni* isolates were tested against nine antimicrobials belonging to six families of antimicrobials selected for being commonly used for the treatment of campylobacteriosis in humans [35,36,37,38]; two quinolones (QNL): ciprofloxacin (CIP, 5 μg) and nalidixic acid (NAL, 30 μg); two β-lactamases (βLAC): ampicillin (AMP, 10 μg) and amoxicillin-clavulanic acid (AMC, 3 μg); two aminoglycosides (AMG): gentamycin (GEN, 10 μg) and streptomycin (S, 10 μg); one macrolide (MCL): erythromycin (E, 15 μg); one tetracycline (TET): tetracycline (TE, 30 μg); and one polymyxin (PMX): colistin (CST, 10 µg) (OXOID antimicrobial susceptibility testing disc dispenser; Antimicrobial Susceptibility Test Disc, OXOID Ltd., England, UK). The source for zone diameters used for interpretation of the test and plates after incubation was the European Committee on Antimicrobial Susceptibility Testing (EUCAST) (http://www.eucast.org/clinical_breakpoints/ accessed on 6 February 2020), and where not possible, according to Clinical and Laboratory Standards Institute (CLSI) indications for *Enterobacteriaceae* (https://clsi.org/media/2663/m100ed29_sample.pdf accessed on 6 February 2020) [39], and the GIDEON guide to antimicrobial agents for CST [40]. The isolates strains were categorised as susceptible (S) or resistant (R), based on EUCAST imperative criteria [41]. Multidrug resistance (MDR) was defined as acquired resistance to at least one agent in three or more antimicrobial classes [12].

### 2.6. Statistical Analyses

A generalised linear model (GLM), which assumed a binomial distribution for *Campylobacter* presence, was fitted to the data to determine whether there was an association between sample type collected (faeces, caeca, carcass, whips, operator, and knives) and *Campylobacter* status of the batch after cooling. A batch was considered infected upon arrival at the slaughterhouse if at least one of the five samples collected from caeca was positive. A batch was considered positive before or after cooling if at least one of the five samples collected from the carcasses was positive. For this analysis, the error was designated as having a binomial distribution, and the probit link function was used. Binomial data for each sample were assigned as one if they were positive for *Campylobacter* or as zero if they were not. Moreover, a GLM was performed to study the relationship between *C. jejuni* and their AMR. A *p*-value of less than 0.05 was considered to indicate a statistically significant difference. Data are presented as least squares means ± standard error of the least squares means. All statistical analyses were carried out using a commercially available software program (SPSS 21.0; SPSS Inc., Chicago, IL, USA).

## 3. Results

**Figure 1:**
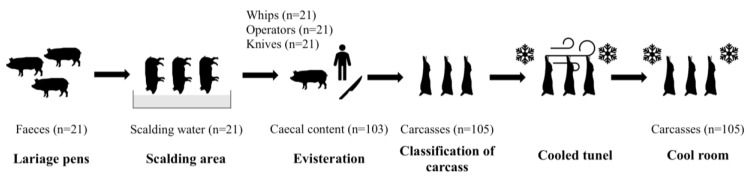
Samples taken during the study.

During this study, a total of 418 samples were collected from different points of the slaughterhouse (Figure 1). Samples were collected from lairage pens (faeces, *n* = 21), caecal content (*n* = 103), carcasses before and after cooling (*n* = 105, respectively), whips (*n* = 21), operators (*n* = 21), working knives (*n* = 21), and scalding water (*n* = 21).

According to the different batches sampled (*n* = 21), all batches that arrived at the slaughterhouse were colonised by *Campylobacter* (caecal samples), and 42.8% (9/21) remained positive after cooling. From all samples collected at the slaughterhouse, 41.9% (175/418) were positive for *Campylobacter,* with all samples collected from scalding water being negative. Thus, the scalding water samples were discarded from the analysis.

Statistically significant differences were observed between the type of sample collected at the slaughterhouse with respect to the percentage of *Campylobacter* isolated (*p-*value < 0.05). Data are represented in Table 1. Regarding to *Campylobacter* speciation, 11 strains from the 175 isolates could not be revived. A total of 41.5% (68/164) strains of *Campylobacter* were speciated into *C. jejuni*, and statistically significant differences were found between the type of sample collected with respect to the percentage of *C. jejuni* isolated (*p-*value < 0.05) (Table 1). Finally, according to an MDR study of *C. jejuni* strains isolated throughout de different slaughter processing steps, no statistically significant differences were observed between the type of sample collected at the slaughterhouse with respect to the MDR *C. jejuni* (*p-*value > 0.05) (Table 1).

### Antimicrobial Susceptibility

From the 68 *C. jejuni* cryovials selected, 54 strains were viable after culture and included in the antimicrobial susceptibility study. All strains analysed were resistant to at least one out of the nine antibiotics tested. The highest percentages of AMR were found to CIP and TE (96.3%, 52/54, both), followed by NAL (88.9%, 48/54), AMP (79.6%, 43/54), and S (77.8%, 42/54). Moreover, the lowest percentages of AMR were found to E (57.4%, 31/54), followed by GEN (27.8%, 15/54), and finally CST (5.6%, 3/54) (*p-*value = 0.000). No resistance was shown against AMC. In addition, statistically significant differences were observed between sample type collected and antimicrobial resistance of *C. jejuni* strains (*p-*value < 0.05) (Table 2).

Furthermore, a total of 96.3% (52/54) *C. jejuni* isolates were resistant to three or more antimicrobial classes (Table 1). Moreover, no significant differences were found between the sample type collected and *C. jejuni* MDR carriage (*p-*value > 0.05).

**Figure 2:**
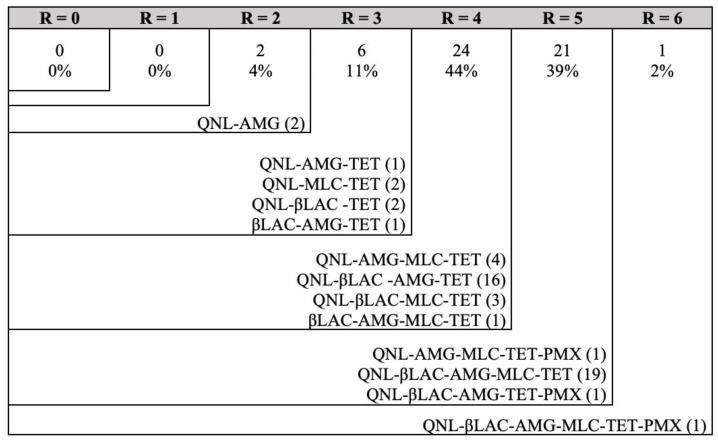
Patterns of resistance for *C. jejuni* isolates. R: number of antibiotic resistances; QNL: quinolones; AMG: aminoglycosides; TET: tetracycline; βLAC: β-Lactamases; MCL: macrolides; PMX: polymyxin. Number within parentheses: number of isolates with the indicated pattern.

Overall, the 13 different resistance patterns observed were summarised in Figure 2. The combination of QNL-βLAC-AMG-MCL-TET (35.2%, 19/54) was the most frequently observed pattern, followed by QNL-βLAC-AMG-TET (29.6%, 16/54). 

## 4. Discussion

The acquisition of MDR is today one of the most important public health concerns. In addition, campylobacteriosis is the main prevalent zoonosis in Europe [10], transmitted mainly by meat consumption. This study demonstrated that MDR *C. jejuni* accounted for 75% of the strains isolated at the animal’s arrival to the slaughterhouse (lairage pens), and at the end of processing 100% of the isolated *C. jejuni* were MDR, constituting a great concern for consumers [42].

The high level of MDR strains detected in this study could be explained due to the absence of a mandatory *Campylobacter* control programme in swine production [43]. Moreover, stressful management practices, such as transport or long stays in lairage pens, may promote higher rates of bacterium increase due to a disturbance in the intestinal functions [17]. In addition, contaminated trucks or contaminated lairage pens from previous batches may induce MDR *C. jejuni* cross-contamination with free-batches [21,44].

In this study, the bacterium was found mainly in swine caecal samples collected. These findings are in agreement with previous results reported by Scanlon et al. [44], with a prevalence of 26% of *Campylobacter* in caecal samples after studying three Irish slaughterhouses. Moreover, there is a strong association between *Campylobacter* status of the batch upon arrival at the slaughterhouse and swine carcass contamination [21]. In this sense, Abley et al. [45] described the association between the contamination of the meat product with the shedding of *Campylobacter* before slaughter. Indeed, the authors found a prevalence of 90% in faeces, which is somewhat higher than our findings (75%) [45]. During evisceration, the carcass contamination could take place by direct contact with intestinal content and could also be re-contaminated after the scalding step [20,22,25]. On the other hand, the percentage of *Campylobacter* carcass contamination before processing was within the range reported previously, from 50 to 100% [31,46]. Nevertheless, previous researches showed higher prevalence of *Campylobacter* in swine carcasses, due to its ability to survive at refrigeration temperatures [45,47]. Moreover, this study showed a significant reduction of *Campylobacter* after cooling, although *C. jejuni* remained constant despite the refrigeration temperature. This fact could be explained because MDR *C. jejuni* has been demonstrated to be more resistant than other *Campylobacter* species, due to its virulence and survival capacity [48].

Although the bacterium is extremely sensitive to the extra-intestinal environment, in this study, it has been isolated from operators, whips, and knives. These results could be explained by the fact that *Campylobacter* has been described as being able to survive adverse condition due to its biofilm forming ability, promoting the maintenance of foodborne pathogens throughout the food chain [20,21,49]. In this sense, a recent study of different Italian swine slaughterhouses showed similar results in the carcass contamination before cooling (50.4%) [31]. The authors of the study related them with the cross-contamination during the slaughter process between batches [31]. Conversely, regarding water samples, Trigui et al. [50] showed water could be an important source of *Campylobacter* cross-contamination in the slaughter line, although our results demonstrated that water is not a risk under our production conditions.

One of the most relevant outcomes of this study is the level of AMR isolated from swine, the most frequently resistance patterns observed being QNL-βLAC-AMG-MCL-TET and QNL-βLAC-AMG-TET. In this line, the latest data from the European Food Safety Authority [12] highlighted the presence of very high levels of resistance against CIP, NA, and TE in *Campylobacter* isolates from humans and pigs, and this situation is notable in Spain. These results are in line with those reported by Mencía-Ares et al. [18], who highlighted the importance of AMR to TET, AMG, and QNL in *Campylobacter* isolates from Spanish swine farms. Although antibiotic treatments these days are controlled in the field, this fact could be explained due to the widespread use of antibiotics in animal production to treat infections in recent years [51,52,53]. In the case of poultry production, Notario et al. [54] found a percentage of resistance for *C. jejuni* below 40%, and in the same line, Rivera et al. [55] observed a resistance below 12%, which highlights the importance of *C. jejuni* and its AMR in the swine production system. This fact is critically important because QNL (e.g., CIP and NA) are drugs of choice for invasive campylobacteriosis infections in humans, and MCL (e.g., E) are the second choice [56], being implicated in reduced treatment effectiveness against *Campylobacter* [56]. Moreover, fluoroquinolone-resistant *Campylobacter* has been included in the WHO priority list of 12 antibiotic-resistant bacteria [7]. In addition, in Spain, levels of *Campylobacter* resistant to E in humans are among the highest in Europe, behind only Portugal and Malta [56]. The same results have been observed in swine production, where these resistances have increased since 2009; however, it should be noted that these data refer to *C. coli* [56].

On the other hand, the level of resistance to GEN and CST in swine *C. jejuni* strains was relatively low. Currently, PMX such as CST represents the last line of defence against resistant severe infections in humans [57]. Thus, it is severely restricted for animal infection treatments, and it is expected that resistance against this antibiotic will decrease in the coming years [58,59].

It is important to highlight that MDRs are important from a public health point of view, but also animal health [60,61]. The high presence of MDR observed at the slaughterhouses also indicates the therapeutic limitations that veterinarians may find on farms to treat common diseases. Furthermore, strains with MDR can lead to horizontal transmission of the resistances between the intestinal microbiota of animals and the environment, causing the MDRs to persist between batches [61,62].

## 5. Conclusions

In conclusion, there is a high level of MDR *C. jejuni* swine batch contamination upon arrival at the slaughterhouse, as well as at the end of the slaughtering process. The difficult control of the bacteria during processing makes it necessary to implement a control programme to reduce the bacterium and their AMR in on-farm and at slaughterhouse level. Further studies are needed to highlight the importance of MDR *C. jejuni* in the swine sector, to assess the bacteria presence in the carcasses, and therefore, to assess the risk for consumers.

## Figures and Tables

**Table 1:** Frequency of *Campylobacter* spp. isolated according to the sample type collected and the relationship with *C. jejuni* and MDR *C. jejuni* isolated.

Sample Type		n_T_	*Campylobacter* spp. (%)	n_c_	*C. jejuni* (%)	n_cj_	MDR *C. jejuni* (%)
Animal samples	Faeces	21	57.0 ± 10.8 ^ab^	12	75.0 ± 12.5 ^a^	8	100 ± 0.0
	Caeca	103	70.0 ± 4.5 ^a^	65	31.0 ± 5.7 ^c^	18	89.0 ± 7.4
	Carcass BC	105	49.0 ± 4.9 ^b^	50	38.0 ± 6.9 ^bc^	14	100 ± 0.0
	Carcass AC	105	27.0 ± 4.3 ^c^	28	54.0 ± 9.4 ^ab^	11	100 ± 0.0
Environmental samples	Whips	21	14.0 ± 7.6 ^c^	3	67.0 ± 27.2 ^abc^	2	100 ± 0.0
	Operator	21	29.0 ± 9.9 ^bc^	5	60.0 ± 21.9 ^abc^	1	100 ± 0.0
	*Knives*	21	14.0 ± 7.6 ^c^	1	0 ^d^	-	-
	*p-value*		<0.001		0.000		>0.05

Data are presented as least squares means ± standard error of the least squares means. n_T_: total samples collected, n_c_: total *Campylobacter* samples speciated, n_cj_: total *C. jejuni* analysed. BC: before cooling, AC: after cooling. MDR: multidrug-resistant. ^a–d^: different superscripts in the same column with uncommon letters are different (*p-*value < 0.05).

**Table 2:** *C. jejuni* antibiotic resistance rates according to the antibiotic and the type of sample collected.

Type of Sample	*n*	CIP	NA	AMP	AMC	GEN	S	E	TE	CST
Faeces	8	87.5	87.5	87.5 ^ab^	0	12.5 ^c^	100 ^a^	62.5 ^b^	100	12.5
Caeca	18	100	88.9	77.8 ^b^	0	22.2 ^bc^	88.9 ^ab^	55.6 ^b^	88.9	5.6
Carcass before cooling	14	92.9	92.9	85.7 ^ab^	0	14.3 ^c^	64.3 ^bc^	42.9 ^b^	100	0
Carcass after cooling	11	100	81.8	72.7 ^b^	0	54.5 ^b^	54.5 ^c^	63.6 ^b^	100	9.1
Whips	2	100	100	50 ^ab^	0	50 ^abc^	100 ^a^	100 ^a^	100	0
Operator	1	100	100	100 ^a^	0	100 ^a^	100 ^a^	100 ^a^	100	0
*p-*value		>0.05	>0.05	0.012	-	0.000	0.000	0.000	>0.05	>0.05

^a–c^: Different superscripts in each column means significant differences with a *p-*value < 0.05. *n*: Number of samples. The resistance was determined by disc diffusion. CIP: ciprofloxacin (5 µg); NAL: nalidixic acid (30 µg); AMP: ampicillin (10 µg); AMC: amoxicillin-clavulanic acid (3 µg); GEN: gentamycin (10 µg); S: streptomycin (10 μg); E: erythromycin (15 μg); TE: tetracycline (30 μg); CST: colistin (10 µg).

## Data Availability

Not applicable.
